# Pregnant Women's Access to PMTCT and ART Services in South Africa and Implications for Universal Antiretroviral Treatment

**DOI:** 10.1371/journal.pone.0027907

**Published:** 2011-12-05

**Authors:** Akthar Hussain, Dhayendre Moodley, Sudhindra Naidoo, Tonya M. Esterhuizen

**Affiliations:** 1 Department of Family Medicine, Nelson R Mandela School of Medicine, University of KwaZulu Natal, Durban, KwaZulu Natal, South Africa; 2 Women's Health and HIV Research Unit, Department of Obstetrics and Gynaecology, Nelson R Mandela School of Medicine, University of KwaZulu Natal, Durban, KwaZulu Natal, South Africa; 3 Programme of Biostatistics, Department of Research Ethics and Medical Law, College of Health Sciences, University of KwaZulu Natal, Durban, KwaZulu Natal, South Africa; University of Witwatersrand, South Africa

## Abstract

**Objectives:**

We describe pregnant womens' access to PMTCT and HAART services and associated birth outcomes in South Africa.

**Methods:**

Women recuperating in postnatal wards of a referral hospital participated in an evaluation during February–May 2010 during which their maternity records were examined to describe their access to VCT, CD4 Counts, dual ART or HAART during pregnancy.

**Results:**

Of the 1609 women who participated in this evaluation, 39% (95%CI36.7–41.5%) tested HIV-positive during their pregnancy. Of the HIV-positive women 2.9% did not have a CD4 count done and an additional 31.3% did not receive their CD4 results. The majority (96.8%) of the HIV-positive women commenced dual ART at their first antenatal visit independent of their CD4 result. During February–May 2010, 48.0% of the women who had a CD4 result were eligible for HAART (CD4<200 cells/mm^3^) and 29.1% of these initiated HAART during pregnancy. Under the current South African PMTCT guidelines 71.1% (95%CI66.4–75.4%) of HIV positive pregnant women could be eligible for HAART (CD4<350 cells/mm^3^). There were significantly more preterm births among HIV-positive women (p = 0.01) and women who received HAART were no more at risk of preterm deliveries (AOR 0.73;95%CI0.39–1.36;p = 0.2) as compared to women who received dual ART. Nine (2.4%; 95%CI1.1–4.5%) HIV exposed infants were confirmed HIV infected at birth. The in-utero transmission rate was highest among women who required HAART but did not initiate treatment (8.5%) compared to 2.7% and 0.4% among women who received HAART and women who were not eligible for HAART and received PMTCT prophylaxis respectively.

**Conclusion:**

In this urban South African community the antenatal HIV prevalence remains high (39%) and timeous access to CD4 results during pregnancy is limited. Under the current South African guidelines, and assuming that access to CD4 results has improved, more than 70% of HIV-positive pregnant women in this community would be requiring HAART.

## Introduction

South Africa, with an estimated 5.6 million people living with HIV, has the largest burden of HIV disease globally [1]. Among these, 330,000 are pediatric HIV infections and are largely due to mother to child transmission. As at mid-2006, during a one year period 38,000 infants were born HIV infected and 26,000 infants in South Africa subsequently acquired HIV during breastfeeding [2]. This was despite the availability of HIV counseling and testing, antiretroviral prophylaxis (single dose NVP) and infant feeding support to prevent mother to child transmission (PMTCT) of HIV in the country.

Given the increasing evidence of the beneficial effects of more than one antiretroviral drug for PMTCT, the National Department of Health in South Africa in 2008 took a decision on commissioning a dual antiretroviral regimen as the standard of care for PMTCT for pregnant women with a CD4 count >200 cells/mm^3^. In addition, pregnant women with a CD4 count <200 cells/mm^3^ were eligible for triple antiretroviral drugs as treatment for themselves (HAART) [3]. The South African PMTCT policy (2008) was introduced to all primary health care clinics in the Umlazi area in February 2008. Women attending antenatal clinics at the primary health centres received HIV counseling and testing. If they tested positive for HIV, women were screened for eligibility for HAART or AZT/NVP as PMTCT prophylaxis based on their CD4 count. Women who were eligible for HAART (CD4<200 cells/mm^3^) at the primary health care clinics were referred to Prince Mshyeni Hospital to commence HAART. HAART was not provided at primary health care clinics. Women with CD4>200 cells/mm^3^ should have received ZDV twice daily (300 mg) from 28 weeks of pregnancy. During labour women should have received a 3 hourly dosing of AZT after taking a single dose of NVP at the onset of labour. HIV exposed infants are routinely tested for HIV from 6 weeks of age by DNA PCR performed on dried blood spots.

There are isolated reports evaluating the implementation of recent PMTCT policy changes ie. HAART for women who are eligible and dual AZT/NVP prophylaxis for those who are not yet eligible for HAART [4], [5]. As opposed to the preceding South African policy of using single dose NVP for PMTCT, it is expected that the complexity of the recent regimens would require well trained staff, well-co-ordinated services and intensive supervision. Hence implementation of new policies should be evaluated in it's infancy to identify protocol deviations that could inevitably become the standard of care if not corrected earlier, and highlight operational challenges that lead to suboptimal coverage and unfavourable outcomes. In May 2010, the South African PMTCT guidelines changed once again to ensure the provision of triple antiretroviral drugs as treatment (HAART) for all HIV positive pregnant women with a CD4<350 cells/mm^3^ [6].

We describe pregnant womens' access to PMTCT and HAART services at primary health care clinics in KwaZulu Natal prior to the implementation of the PMTCT policy change in May 2010. We also compare birth outcomes such as preterm delivery, stillbirths, low birth weight and inutero HIV transmission and neonatal morbidity in 3 categories of women viz. women who received ART prophylaxis for PMTCT (HAART ineligible), women eligible for HAART but did not commence HAART (HAART eligible/untreated) and women who were eligible for and commenced HAART (HAART eligible/treated).

## Methods

This is a cross-sectional evaluation of PMTCT and HAART services and birth outcomes in Umlazi, the second largest township in South Africa with an estimated HIV antenatal prevalence of 40%. The evaluation conducted postnatally comprised of a maternity chart audit conducted in the post delivery wards of Prince Mshiyeni Memorial Hospital during a four month period between February and May 2010. Prince Mshiyeni Hospital is a District/Regional Hospital, supports 17 primary health care clinics and has an annual birth rate of 12,000.

A written informed consent from all potential participants was obtained prior to any research activity. The informed consent (Zulu and English) were administered by two trained research assistants. Maternity records of consenting study participants were examined to describe their antenatal attendance, access to voluntary counseling and testing and access to CD4 Counts, AZT/NVP and HAART. Birth outcomes such as stillbirth rate, Low birth weight rate, preterm delivery rate and inutero HIV transmission rates were compared between the 3 categories of women viz. HAART ineligible, HAART eligible/untreated and HAART eligible/treated. A dried blood spot was collected from a subsample of HIV exposed infants at birth for HIV diagnosis by DNA PCR.

The following data were collected by chart review:

- Demographic and Antenatal Attendance to include Gestational Age at Booking, Gravidity, Marital Status, Age and Number of Antenatal Visits
- VCT, PMTCT and ARV access to include record of VCT access, HIV test results, access to CD4 investigation, CD4 result, eligibility for HAART, access to HAART or PMTCT ARV prophylaxis (AZT/NVP).
- Obstetric and Birth Outcomes to describe Birth Outcome (LiveBirth/MSB/FSB), Gestational age at birth, and Birth Weight.

Stata version 10 (StataCorp, Texas, U.S.A) was used to analyse the data. A general descriptive analysis was conducted to address all objectives using median, mean, range and 95%CI where applicable. Maternal characteristics presented as categorical data were compared using the Pearson's chi square test. A multivariate analysis was performed in determining independent associations between birth outcomes, HIV status and exposure to HAART. A p value of <0.05 was considered statistically significant. Data were analysed according to stratification of the study population with CD4<200 cells/mm^3^, CD4>200 cells/mm^3^, HAART ineligible, HAART eligible/untreated and HAART eligible/treated.

The study was approved by the Ethics Committee of the Nelson R Mandela School of Medicine. All patient details remained confidential and a written informed consent was obtained from eligible participants.

## Results

**Figure 1:**
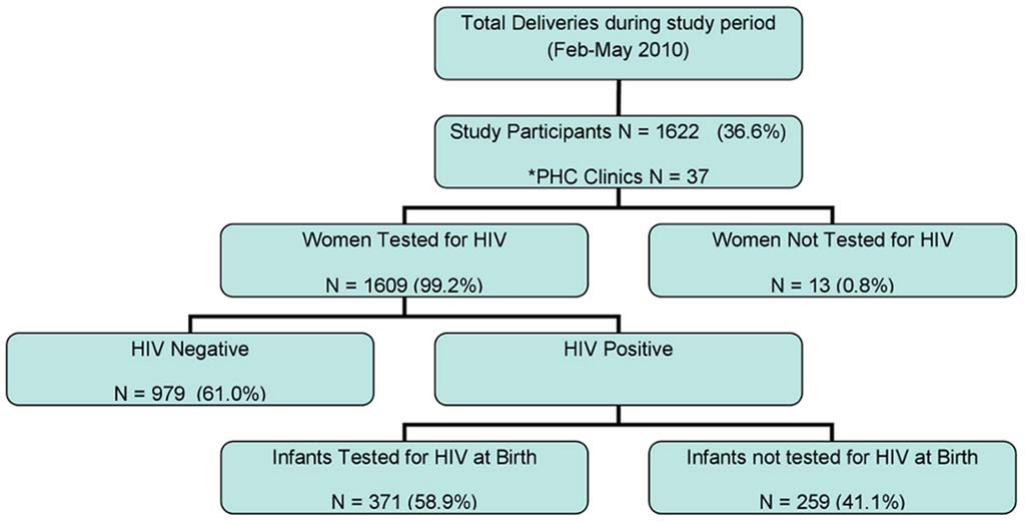
Study Population Included in the PMTCT/ART Service Evaluation. *PHC – Primary Health Care Clinics.

A total of 1622 women participated in this postnatal audit; 36.6% of the total deliveries at this regional hospital for the period February to May 2010 (Figure 1). The maternity department of the hospital recorded an average of 35 births a day and we evaluated an average of 16 participants per day. According to their antenatal records, the women who delivered at Prince Myshyeni Hospital during the 4 month study period received antenatal care at any one of the 37 primary health care (PHC) clinics in the catchment area. The study sample therefore included a representation of all primary health care facilities in the Umlazi area.

One thousand, six hundred and nine (99.2%) women who participated in the audit were tested for HIV at their respective PHC clinics during their antenatal care; 630 (39%; 95%CI 36.7–41.5) tested positive for HIV (Figure 1).

The median age (±SD) of this study population was 24 (±6.1) years; half the population were 24 years or younger (Table 1). One in 3 women was pregnant for the first time (32%), and the majority (68%) had 2 children or more including this recent birth. The majority of women (88.8%) were single and not living with their partner. Women registered for antenatal care at a median gestational age of 23, ranging from 4 to 39 weeks (Table 1). Two-thirds (74.3%) of the women registered between 14 and 28 weeks in pregnancy and 1.2% registered >36 weeks in gestation. Demographic and antenatal registration characteristics did not differ between the HIV positive and negative groups of women.

**Table 1:** General Characteristics of Study Population n = 1622.

	N	%	Min	Max	Median(SD)
**Age (years)**			12	50	24(6.1)
24 or younger	852	52.5			
25 or older	770	47.5			
**Marital Status**					
Single and Not Living with Partner	1441	88.8			
Married	112	6.9			
Single and Living with Partner	4	0.3			
Other	65	4.0			
**Gravidity**			1	11	2(1.1)
Single	519	32.0			
Multiple	1,103	68.0			
**Gestational Age at Antenatal Registration**			4	39	23(6.2)
<14 wk	115	7.1			
14–28 wk	1196	74.3			
29–36 wk	217	13.5			
>36 wk	19	1.2			
**HIV Status**					
HIV+	630	39.0%			
**CD4 count (cells/mm^3^)**	415		13	1228	285(IQR 139–392)
**Birth Weight**	1564		0.6	5.3	3.1(0.59)
LBW (<2500 g)	232	14.8%			
Normal	1,331	85.2%			
**Birth Outcome**					
Live Birth	1,562	97.0%			
Still Birth (MSB+FSB)	48	3.0%			

**Figure 2:**
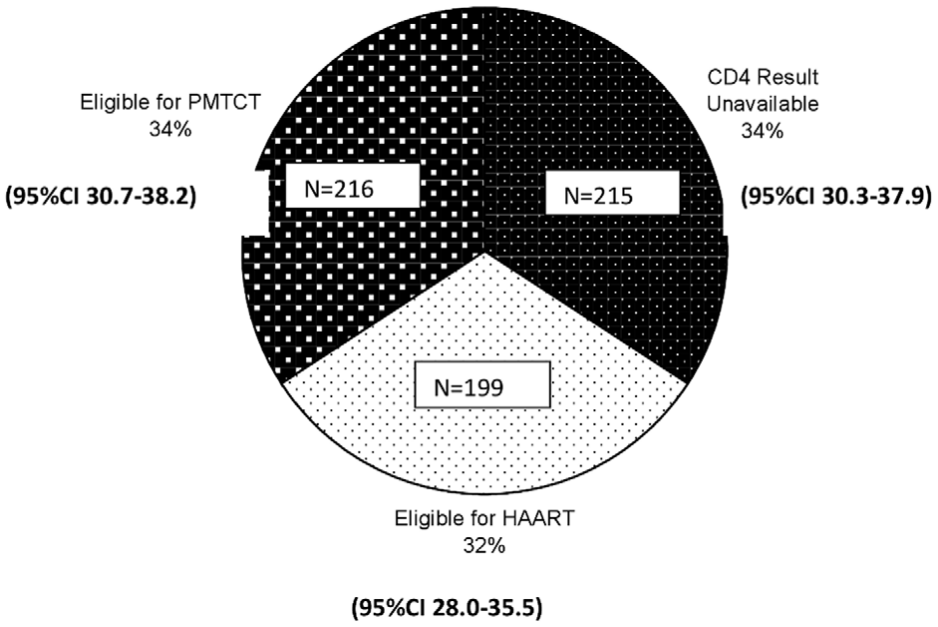
Women's Access to Dual ART regimen for PMTCT or HAART as treatment based on the availability of CD4 results and CD4 Count<200 (n = 630).

**Figure 3:**
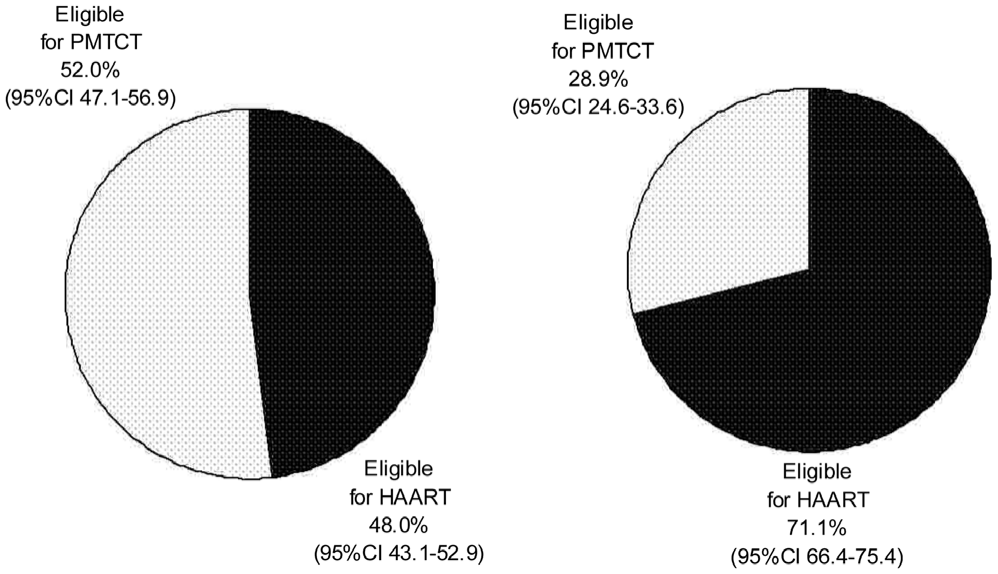
Using CD4 Count to guide clinical management of HIV positive pregnant women (n = 630).

Among the 630 women with a positive HIV status, 97.3% had a CD4 test performed. CD4 results were unavailable for 34.2% of women (2.9% did not have a CD4 count done and 31.3% did not receive their results (Figure 2). The median CD4 count for this population was 285 (IQR 139–392) cells/mm^3^. Among the women who had a CD4 result 199 (48% 95%CI 43.1–52.9) were eligible for HAART according to the 2008 PMTCT guidelines (Figure 3). Six hundred and ten (96.8%) of the HIV positive women initiated dual ART prophylaxis for PMTCT independent of their CD4 result; and 58 (9.2%) of of all HIV positive pregnant women initiated HAART. Among the women eligible for HAART (CD4<200 cells/mm^3^), 29.1% initiated HAART during their pregnancy and 18 (2.9%) of HIV positive pregnant women did not receive any antiretroviral drugs for PMTCT or treatment. Among the women who had a CD4 result, 295 (71.1%; 95%CI 66.4–75.4) had a CD4<350 cells/mm^3^ and would have been eligible for HAART according to the current (2010) PMTCT guidelines (Figure 3).

There were 352 (22.0%) preterm births in the study population (n = 1622). The median gestational age at delivery in the preterm and term groups were 35 (16.5–37) and 38 (38–40) weeks respectively. There was a significantly higher proportion of preterm births among the HIV positive women (25.20% vs 19.78%; p = 0.01) (Table 2). Women who received HAART were no more at risk of preterm deliveries as compared to their counterparts who were eligible but did not receive HAART (AOR 0.73; 95%CI 0.39–1.36; p = 0.2) (Table 3).

**Table 2:** Birth Outcomes among HIV positive and Negative women.

	HIV PositiveN = 630	HIV NegativeN = 928	OR (95%CI)	P value
**Gestational Age at Delivery** *				
**<37 weeks**	158 (25.20%)	194 (19.78%)		
**>37 weeks**	469 (37.3%)	787 (62.7%)	1.37 (1.08–1.74)	0.01
**Birth Outcome**				
**Stillbirths**	17 (2.73%)	31 (3.17%)		
**Live Births**	605	945	0.91 (0.5–1.6)	0.43
**Birthweight**				
**Median±SD**	3.1±0.50	3.1±0.47		
**<2500 g**	43 (9.4%)	42 (5.4%)		
**≥2500 g**	587	886	1.56 (1.18–2.07)	0.002

*Gestational Age at Birth was calculated using the Ballard Score.

**Table 3:** Birth Outcomes among HIV positive women who received or did not receive PMTCT prophylaxis or HAART (eligibility based on available CD4 count).

	PMTCT Eligible and Received PMTCT AZT/NVP* (n-216)	HAART Eligible and received HAART (n = 58)	HAART Eligible but did not receive HAART (n = 141)	Total N = 415	Adjusted Odds Ratio (95%CI)	p value
**Preterm**	2 (1.6%)	34 (26.6%)	92 (71.9%)	128 (31.1%)	0.73 (0.4–1.4)	0.20
**Term**	212	24	48	284		
**Stillbirths**	1 (9.0%)	1 (9.0%)	9 (81.8%)	11 (2.7%)	0.23 (0–1.4)	0.11
**Live Births**	213	57	131	401		
**Birthweight <2500 g**	3 (27%)	4 (36.4%)	4 (36.4%)	11 (2.7%)	1.03 (0.4–2.0)	0.53
**Birthweight ≥2500 g**	211	54	136	401		

*Only 2 women did not receive PMTCT prophylaxis AZT/NVP. Excluded all other women who received PMTCT prophylaxis but their eligibility for HAART was unknown (CD4 count unknown).

Forty eight (3.0%) still births were reported for the study population, and the stillbirth rate did not differ significantly between the HIV positive and negative groups (28.5/1000 births vs 33.4/1000 births) (p = 0.43) (Table 2). Maternal exposure to HAART as treatment did not influence the still birth rate in this population (AOR 0.23; 95%CI 0–1.4; p = 0.11) (Table 3), although macerated still births were more common among women who were eligible for HAART and did not initiate HAART as compared to their counterparts who initiated HAART (64/1000 births vs 17/1000 births). This association was not statistically significant (p = 0.28).

Excluding the preterm births, the mean (± SD) birthweight of children born was 3.1±0.59; 232 (14.8%) of the infants weighed <2500 g at birth (LBW). LBW was significantly more common among HIV positive women (p = 0.002) (Table 2). Exposure to HAART as treatment did not significantly influence birth weight (AOR 1.03; 95%CI 0.4–2.0; p = 0.53 (Table 3)).

Three hundred and 71 (58.9%) of the 630 HIV exposed infants were tested for HIV by DNA PCR at birth; 9 of these infants were confirmed HIV infected (2.4%; 95%CI 1.1–4.5) at birth. Eight of the 9 (88.9%) infants were born to women with CD4 count ranging between 13 and 41. None of these women initiated HAART despite being eligible for treatment. The in-utero transmission rate was highest among women who required HAART but did not initiate treatment (8.5%) compared to 2.7% and 0.4% among women who received HAART and women who were not eligible for HAART and received PMTCT prophylaxis respectively.

## Discussion

A year ago (2010) under the previous South African policy for the management of HIV pregnant women with a CD4 count <200 cells/mm^3^, a third (34%) of HIV positive pregnant women in a large urban community did not receive their CD4 results during pregnancy and hence missed the opportunity of being referred for HAART. Of greater concern, only 29% of the HIV positive pregnant women who required HAART actually initiated HAART during pregnancy. The impact of not receiving HAART was seen in the 3 fold higher incidence of inutero HIV transmission among women who were HIV positive, with a CD4<200 cells/mm^3^ and did not receive HAART.

Since South Africa revised their guidelines on the use of ARV for PMTCT in 2010, a shift in the eligibility criteria of CD4<350 cells/mm^3^ would translate to 71% of the current HIV positive pregnant women in this South African study population being eligible for HAART [6]. In a Zambian cohort of pregnant women, 54% of women had a CD4 count <350 cells/mm^3^ and a larger proportion (68%) met the new WHO treatment criteria if clinical stage III in addition to CD4 count <350 cells/mm^3^ were considered [7], [8]. The new guidelines WHO were based on overwhelming evidence with an intent to improve the mother's health while ensuring optimal prevention of MTCT [7].

Commencing pregnant women with CD4<350 cells/mm^3^ with HAART could prevent more than 75% of MTCT not only during pregnancy but through breastfeeding as well [9]–[11]. Our study demonstrated a 3 fold higher in-utero transmission rate (8.5%) among women who required HAART (CD4<200 cells/mm^3^) and did not receive this treatment as compared to those who were eligible and received treatment. Historically, in utero transmission accounted for 20–30% of vertical transmission of HIV in the absence of an intervention [12]. In the PEPI study in Malawi, in utero HIV transmission was reportedly 6.7% but higher (17.8%) among women with incident HIV infections in pregnancy [13]. Yet another reason for HAART in all pregnant women who test HIV positive independent of a CD4 count. The BAN study conducted in Malawi reported a 4.9% in-utero transmission [11]. In the Kesho Bora study, lower in utero transmission was significantly associated with undetectable maternal viral load at delivery and in the presence of triple antiretroviral (2.5% vs 1.8%) as opposed to ZDV and single dose NVP [10].

The latest recommendation underscores the use of HAART for HIV positive pregnant women with CD4<350 cells/mm^3^ based on the overwhelming evidence of a strong association between HIV and maternal deaths in resource limited countries with a high HIV burden. Maternal mortality rate in South Africa for the 2005–2007 triennium was reported as 152 per 100,000 deliveries, and AIDS contributed to 43.7% of these maternal deaths [14]. Pregnancy related sepsis remains the 4^th^ commonest cause of maternal deaths and most likely related to HIV and an immunocompromised state. According to this “Saving Mother” report, pregnant women who are HIV infected are 10 times more likely to die in the pueperium period as compared to HIV uninfected women (MMR 327.7/100,000 vs 34.4/100,000). Increasing the CD4 threshold for HAART in South Africa was therefore much needed to reduce HIV related maternal mortality.

Several major studies present conflicting evidence of a link between preterm deliveries and exposure to HAART during pregnancy [15], [16]. Other studies have implicated the use of protease inhibitors in increased risk of preterm births [17], [18]. A South African study reported a significantly higher premature birth rate of 5% in HAART unexposed women vs 14% in HAART exposed women [19]. The investigators concluded that early HAART exposure was strongly associated with an increased risk for preterm births. However, the study population only comprised of immunocompromised women with a CD4 count <250 cells/mm^3^ [19]. Our conclusions are inconsistent with the latter study. We demonstrated a higher proportion of preterm births among women who required HAART (CD4<200 cells/mm^3^) independent of HAART exposure. There was no added effect of HAART exposure, hence providing further evidence that women who require HAART (severely immunocompromised) are at a higher risk of preterm births as compared to their counterparts with a CD4>200 cells/mm^3^ (not yet immunocompromised). Previous studies have not had an opportunity to compare the occurrence of preterm births in women eligible for and did not receive HAART as compared to their counterparts who received HAART or women with CD4>200 cells/mm^3^. In a pooled analysis comparing highly active antiretroviral therapy with dual regimens for PMTCT, Townsend et al reiterate an association with preterm delivery and HAART but also propose that conflicting reports of such an association between HAART and preterm births could be due to confounding determinants, variability in study design, population variability and differences in data collected [20]. Our study adds to a list of previous studies demonstrating no link between HAART and preterm births, and further supports a stronger association between preterm birth and advanced HIV disease stage independent of HAART.

Undoubtedly, there are concerns to offering HAART to 30% women who may not yet require HAART that include side effects such as anemia and hepatotoxicity. The safety of triple ART drug regimens in pregnant women with CD4>350 and the effect of discontinuing such regimens on maternal health remains to be confirmed.

We also agree that there are major economical and logistical challenges in expanding HIV treatment programmes in resource-limited countries [2], [21], [22]. Our study highlights one of these many challenges in receiving CD4 results timeously to commence HAART early. A third of the study population who had a CD4 done during pregnancy, did not have a CD4 result at the time of delivery. In such a setting with a high antenatal HIV prevalence (39%), and more than two thirds (71%) of women eligible for HAART with a CD4<350 cells/mm^3^, it would be logical/prudent to consider the “Test and Treat” principle to ensure all pregnant women who are HIV infected receive HAART early in pregnancy. Alternatively, in the absence of reliable access to CD4 testing or the timeous availability of CD4 results, clinical staging has been proposed as an alternative indication for HAART. However, the sensitivity of clinical staging in identifying patients in need for HAART has been reportedly low as compared to CD4 results (23% vs 94%) in the multi-country MTCT-Plus Initiative [23]. Given the overwhelming evidence of benefits of initiating HAART early in pregnancy and the vast majority of women eligible for HAART we are more inclined to support the recommendations proposed by Zolfo et al for universal initiation of HAART in all HIV positive pregnant women irrespective of CD4 count [24].

As predicted, the growing case load of patients requiring HAART over time has posed numerous challenges to central facilities that provide HAART services in the public health sector. In our study population, women were referred to a central HAART service at a hospital, the only one that supported 17 such PHC clinics in the area. Women who were referred for HAART to the central service often returned to the PHC for continued antenatal care without registering for HAART. The lack of human resources at the centralised HAART services, distance between PHC clinics and HAART services, poor referral mechanisms and non-existent links between PHC and the central service are plausible reasons for the vast majority of women not initiating HAART in pregnancy. Based on the above arguments, countries have been encouraged to decentralise HAART services to PHC facilities to ensure wide scale coverage. Nursing personnel currently providing and monitoring PMTCT prophylaxis in pregnant women would require additional training in modifying their management protocols to include HAART. A combination of differential management protocols for women in the varying stages of the disease and a lack of effective monitoring systems at a PHC also pose a challenge to an already overburdened human resource system.

There are several limitations to the study. Access to HIV clinical services was based on a retrospective assessment of documentation in medical records, and these sources of information could not be verified. Information related to the nature and actual commencement of the HAART regimens as well as other obstetric risk factors were limited. This study was conducted in a single urban community and may not be representative of the larger South African population.

In conclusion, if more than 70% of HIV positive pregnant women in an urban South African community are requiring HAART under the current South African guidelines, and access to CD4 count remains limited, the country's HIV management policy for pregnant women needs to be modified. The high HIV antenatal prevalence (39%), the majority (70%) of women being eligible for HAART (CD4<350 cells/mm^3^) and the small proportion of women actually initiating HAART at a referral centre are all compelling reasons for initiating HAART in all HIV positive pregnant women at primary health clinics until after delivery or until breastfeeding ceases and there is no further risk of MTCT. Primary health clinics do not have laboratory services and may often lack good communication links with regional laboratories, hence the limited access to CD4 results would prevent the vast majority of HIV positive pregnant women from receiving HAART.
